# Genetic deletion of MMP12 ameliorates cardiometabolic disease by improving insulin sensitivity, systemic inflammation, and atherosclerotic features in mice

**DOI:** 10.1186/s12933-023-02064-3

**Published:** 2023-11-28

**Authors:** Melina Amor, Valentina Bianco, Martin Buerger, Margarete Lechleitner, Nemanja Vujić, Anja Dobrijević, Alena Akhmetshina, Anita Pirchheim, Birgit Schwarz, Ariane R. Pessentheiner, Franziska Baumgartner, Katharina Rampitsch, Silvia Schauer, Iva Klobučar, Vesna Degoricija, Gudrun Pregartner, Daniel Kummer, Monika Svecla, Gerhard Sommer, Dagmar Kolb, Gerhard A. Holzapfel, Gerald Hoefler, Saša Frank, Giuseppe Danilo Norata, Dagmar Kratky

**Affiliations:** 1 Gottfried Schatz Research Center, Molecular Biology and Biochemistry, Medical University of Graz, Neue Stiftingtalstrasse 6/4, Graz, 8010 Austria; 2 Institute for Vascular Biology, Center for Physiology and Pharmacology, Medical University of Vienna, Vienna, Austria; 3 Institute for Molecular Biosciences, University of Graz, Graz, Austria; 4 Institute of Biomechanics, Graz University of Technology, Graz, Austria; 5 Diagnostics and Research Institute of Pathology, Medical University of Graz, Graz, Austria; 6 Sisters of Charity, University Hospital Centre, Zagreb, Croatia; 7 University of Zagreb School of Medicine, Zagreb, Croatia; 8 Department of Medicine, Sisters of Charity, University Hospital Centre, Zagreb, Croatia; 9 Institute for Medical Informatics, Statistics and Documentation, Medical University of Graz, Graz, Austria; 10 Gottfried Schatz Research Center, Cell Biology, Histology and Embryology, Medical University of Graz, Graz, Austria; 11 Department of Pharmacological and Biomolecular Sciences, University of Milan, Milan, Italy; 12 Core Facility Ultrastructural Analysis, Medical University of Graz, Graz, Austria; 13 BioTechMed-Graz, Graz, Austria; 14 Department of Structural Engineering, Norwegian University of Science and Technology, Trondheim, Norway

**Keywords:** CMD, Matrix metalloproteinase 12, MMP12 deficiency, Ldlr-deficient mice, Proteomics, Metabolic syndrome patients

## Abstract

**Background:**

Matrix metalloproteinase 12 (MMP12) is a macrophage-secreted protein that is massively upregulated as a pro-inflammatory factor in metabolic and vascular tissues of mice and humans suffering from cardiometabolic diseases (CMDs). However, the molecular mechanisms explaining the contributions of MMP12 to CMDs are still unclear.

**Methods:**

We investigated the impact of MMP12 deficiency on CMDs in a mouse model that mimics human disease by simultaneously developing adipose tissue inflammation, insulin resistance, and atherosclerosis. To this end, we generated and characterized low-density lipoprotein receptor (Ldlr)/Mmp12-double knockout (DKO) mice fed a high-fat sucrose- and cholesterol-enriched diet for 16–20 weeks.

**Results:**

DKO mice showed lower cholesterol and plasma glucose concentrations and improved insulin sensitivity compared with LdlrKO mice. Untargeted proteomic analyses of epididymal white adipose tissue revealed that inflammation- and fibrosis-related pathways were downregulated in DKO mice. In addition, genetic deletion of MMP12 led to alterations in immune cell composition and a reduction in plasma monocyte chemoattractant protein-1 in peripheral blood which indicated decreased low-grade systemic inflammation. Aortic *en face* analyses and staining of aortic valve sections demonstrated reduced atherosclerotic plaque size and collagen content, which was paralleled by an improved relaxation pattern and endothelial function of the aortic rings and more elastic aortic sections in DKO compared to LdlrKO mice. Shotgun proteomics revealed upregulation of anti-inflammatory and atheroprotective markers in the aortas of DKO mice, further supporting our data. In humans, MMP12 serum concentrations were only weakly associated with clinical and laboratory indicators of CMDs.

**Conclusion:**

We conclude that the genetic deletion of MMP12 ameliorates obesity-induced low-grade inflammation, white adipose tissue dysfunction, biomechanical properties of the aorta, and the development of atherosclerosis. Therefore, therapeutic strategies targeting MMP12 may represent a promising approach to combat CMDs.

**Supplementary Information:**

The online version contains supplementary material available at 10.1186/s12933-023-02064-3.

## Background

Cardiometabolic diseases (CMDs) represent a group of interrelated conditions that include visceral obesity, type 2 diabetes (T2D), dyslipidemia, hypertension, non-alcoholic fatty liver disease, and cardiovascular disease (CVD) [1]. The rising prevalence of obesity during the past decades has become a major driver for the development of CMDs, turning them into a substantial biomedical and socioeconomic challenge [2, 3]. An urgent need to tackle the metabolic and cardiovascular risk factors associated with obesity has prompted identification of common mechanisms as the most promising therapeutic approach against these diseases [4]. Among others, chronic low-grade systemic inflammation has been recognized as an underlying cause that links the different pathologies of CMDs, primarily insulin resistance and atherosclerosis [5–7]. Blockade of key factors contributing to chronic inflammation in metabolic and vascular tissues therefore represents a potential therapeutic approach to jointly address insulin resistance and atherosclerosis, the underlying causes of T2D and CVD, respectively.

In an unbiased bioinformatic approach combining published human data, microarray analyses of mouse white adipose tissue (WAT) and aorta, and a meta-analysis, we identified MMP12 as a massively upregulated pro-inflammatory factor in metabolic and vascular tissues in mice and humans suffering from CMDs [8]. MMP12 belongs to the MMP family, which comprise 23 zinc-dependent endopeptidases that share a common multidomain structure (9). Traditionally, MMPs were associated with extracellular matrix (ECM) degradation and turnover and thus tissue remodeling [10]. Recently, MMPs have also been shown to play critical roles in a variety of biological processes, including metabolism and inflammation [11]. MMP12 is a macrophage-secreted protein with elastin as its preferred substrate that is involved in the onset of chronic inflammation affecting neutrophil infiltration, cytokine release, macrophage recruitment, and proliferation [12–15]. Accordingly, the enzyme has been proposed as a potential target for a variety of inflammatory conditions, including respiratory diseases [16], granulomas [17, 18], different types of cancer [19–21], and neurological and musculoskeletal disorders [22, 23]. Knockdown of MMP12 in the small intestine ameliorated high-fat diet-induced metabolic disturbances and intestinal homeostasis in mice [24]. Moreover, the enzyme also plays a deleterious role in obesity-induced chronic kidney disease by aggravating glomerular fibrogenesis, oxidative stress, and inflammation [25]. Additionally, several studies using different animal models of adipose tissue inflammation and insulin resistance or atherosclerosis have indicated a pathological role of MMP12 in T2D and CVD [26–30]. However, its involvement in insulin resistance and WAT dysfunction remains inconsistent and requires further clarification [31, 32], and its role in obesity-induced low-grade systemic inflammation and vascular homeostasis is still unclear.

We therefore investigated the consequences of genetic deletion of MMP12 on the Ldlr-deficient background (DKO) in mice fed a high-fat sucrose- and cholesterol-enriched (HFSC) diet that mirror human disease by simultaneously developing the complications associated with CMDs, such as WAT inflammation, insulin resistance, and cardiovascular dysfunction [33]. We demonstrated that genetic knockout of MMP12 alleviates CMDs in mice and that circulating MMP12 levels in humans may serve as a diagnostic marker for CMDs. Our study suggests that therapeutic strategies targeting MMP12 may be a promising approach against the development or progression of CMDs.

## Methods

### Animals and diet

Mmp12KO were crossed with LdlrKO mice (both from The Jackson Laboratory, Bar Harbor, ME) to generate LdlrMmp12-DKO mice. Age- and sex-matched LdlrKO and DKO mice were housed in a clean and temperature controlled-environment (22 ± 1 °C; relative humidity 45–65%) with unlimited access to food and water on a regular 12-h/12-h light–dark cycle. At 6 weeks of age, female and male mice were fed a HFSC diet (0.18% cholesterol, 58 kcal% fat (primarily lard), 28 kcal% carbohydrates (with 17.5 kcal% from sucrose); D09071704, Research Diets Inc, New Brunswick, NJ) for 20 and 16 weeks, respectively. Body weight was monitored weekly during dietary treatment. Female mice were used for atherosclerosis, whereas male mice were used for metabolic studies. All experiments were performed in accordance with the European Directive 2010/63/EU and approved by the Austrian Federal Ministry of Education, Science and Research (Vienna, Austria; BMBWF-66.010/0138-V/3b/2019).

### Plasma lipid parameters and lipoproteins analysis

Blood was collected by facial vein puncture from 12-h fasted mice. Lipoprotein fractions were separated using 200 µl of pooled plasma per genotype. Plasma lipid parameters (triglycerides (TG), total cholesterol (TC), free cholesterol (FC), cholesteryl esters (CE)) and lipoprotein profiles were determined as previously described [34].

### Plasma glucose measurements

Blood glucose concentrations were measured in 12-h fasted mice using Accu-Chek® Active glucometer and glucose strips (Roche Diagnostics, Basel, Switzerland)

### Glucose tolerance test (GTT) and insulin tolerance test (ITT)

For the GTT, mice were fasted for 6 h and then gavaged with 2 g glucose/kg body weight. For the ITT, mice were fasted for 5 h and then injected i.p. with 0.5 IU insulin/kg body weight (Actrapid; Novo Nordisk, Bagsværd, Denmark). In both tests, blood glucose concentrations were determined 0, 15, 30, 60, 90, and 120 min after glucose or insulin administration using Accu-Chek® Active glucometer and glucose strips (Roche Diagnostics, Basel, Switzerland).

### RNA isolation and quantitative real-time PCR analysis

Total RNA was isolated using TRISure™ reagent (Meridian, Memphis, TE) according to the manufacturer’s protocol. Two µg of total RNA were reverse transcribed into cDNA using the High-Capacity cDNA Reverse Transcription Kit (Thermo Fisher Scientific, Waltham, MA). Quantitative real-time PCR was performed on a CFX96 Real-Time PCR detection system (Bio-Rad Laboratories, Hercules, CA) using the GoTaq® qPCR Master Mix (Promega, Madison, WI). Samples were normalized to RPLP0 mRNA expression as reference gene. Expression profiles and associated statistical parameters were determined by the 2^−ΔΔCT^ method. Primer sequences are listed in Additional file 1, Table S1.

### Western blotting

One hundred mg of epididymal WAT (eWAT) were lysed in RIPA buffer supplemented with protease/phosphatase inhibitor cocktail (PIC) (1: 1,000; Merck, Darmstadt, Germany). Subsequently, the samples were centrifuged at 16,000 x *g* and 4 °C for 30 min, the middle fat-free layer was isolated, and protein concentrations were estimated (DC™ Protein assay, Bio-Rad Laboratories, Hercules, CA). Fifty µg of protein were separated by SDS-PAGE and transferred to a PVDF membrane. The blot was incubated with rabbit polyclonal anti-BAX (1:1,000, #2772) and anti-calnexin (CNX) antibodies (1:1,000, #2679T) (both Cell Signalling Technology, Danvers, MA). HRP-conjugated goat anti-rabbit antibody (1:2,500, #31,460; Thermo Fisher Scientific, Waltham, MA) was visualized by enhanced chemiluminescence detection on a ChemiDoc™ MP imaging system (Bio-Rad Laboratories, Hercules, CA). BAX was quantified by densitometry (ImageJ® Software, Version 1.51) and normalized to the expression of CNX.

### Histology and F4/80 immunohistochemistry (IHC) and quantification of F4/80-positive regions

eWAT samples were fixed in 4% neutral-buffered formalin for 24 h and subsequently embedded in paraffin. Section (7 μm) were deparaffinized and stained with hematoxylin and eosin (H&E) as described elsewhere [35]. Immunohistochemical staining was performed by incubating the sections with anti-mouse F4/80 antibody (1:50, #MCA497G; Bio-Rad, Hercules, CA) for 1 h at RT. Thereafter, the sections were washed three times with PBS, incubated for 30 min with biotinylated polyclonal rabbit anti-rat immunoglobulin (1:100, #E0468; Dako, Glostrup, Denmark), and washed three times with PBS. To visualize antibody binding, sections were incubated with AEC substrate (#K3464; Dako) for 5–30 min at RT. Nuclei were counterstained with Mayer’s hematoxylin for 30 s, washed with tap water, and mounted with aqueous mounting agent (#1.08562; Merck, Darmstadt, Germany). Images were acquired at 20–40X magnification using an Olympus BX63 microscope equipped with an Olympus DP73 camera (Olympus, Shinjuku, Japan). White adipocyte size was quantified using the automated ImageJ plugin Adiposoft [36].

To quantify F4/80-positive regions, images were analyzed using Visiopharm (version 2021.09). Tissue regions were manually labeled, and color deconvolution was performed for F4/80 staining, resulting in an image of staining intensity to which a threshold for F4/80 positivity was applied. The cumulative F4/80 staining intensities were used to calculate the mean F4/80 intensity on the tissue regions within one image.

### Untargeted proteomics analysis of aorta and eWAT

#### Sample preparation

Aortic arch and descending thoracic segments from LdlrKO and DKO mice (n = 6/group) were pooled up to 20 mg and homogenized in in the presence of RIPA buffer (Tris-HCl 50 mM pH 7.2, EDTA 5 mM, SDS 0.1%) supplemented with protease inhibitors at a ratio of 1:100 (# 5872 S, Cell Signaling, Danvers, MA) as previously described [37]. eWAT from each genotype (n = 6/group) was pooled up to 100 mg and processed for proteomic analysis as previously described, with minor modifications [38, 39]. Briefly, fat pads were sonicated 2 × 10 s in a buffer containing 50 mM HEPES, 1% Triton, 100 mM NaF, 10 mM Na-orthovanadate, 10 mM EDTA, 0.2% SDS, 100 mM NaCl, with 30 s break on ice. Homogenates were processed at 4 °C using Tissue Ruptor (QIAGEN, Venlo, Netherlands) for 5 min, cycle 30/30 s. Proteins were separated from fat and tissue debris by centrifugation at 20,000 *x g* and 4 °C for 30 min. Thereafter, proteins were precipitated with four times the volume of acetone per sample and incubated for 15 min at -80 °C and 120 min at -20 °C. Proteins were pelleted by centrifugation at 16,000 *x g* and 4 °C for 15 min, then air-dried and resuspended in buffered urea (8 M, Tris-HCl 0.1 M, pH 8.5). The protein content in eWAT and the aorta was determined for all tissues using a Lowry assay. A volume corresponding to 10 µg of proteins from aorta and 50 µg of proteins from eWAT was completely dried in a vacuum concentrator at 45 °C for 45 min. The dried protein pellet was resuspended in 10 µl of water with the addition of 10 µl of ammonium bicarbonate (50 mM, pH 8.5), followed by protein reduction with 100 mM DTT for 30 min at 55 °C. Proteins were then alkylated at RT by incubation with 150 mM iodoacetamide in 50 mM ammonium bicarbonate solution for 30 min in the dark. Trypsin digestion (#T7575-1KT; Merck, Darmstadt, Germany) with a 1:20 enzyme to protein ratio was performed overnight at 37 °C and terminated by acidification with 1% trifluoroacetic acid. LC-MS/MS analyses were performed as previously described [40]. In brief, samples were analyzed in duplicate using a Dionex Ultimate 3000 nano-LC system (Dionex, Sunnyvale, CA) connected to an Orbitrap Fusion™ Tribrid™ Mass Spectrometer (Thermo Fisher Scientific, Waltham, MA) operating in positive ion mode with a nanoelectrospray ion source. The peptide mixtures were preconcentrated on an Acclaim PepMap column (C18, 100 Å, 100 μm ID x 2 cm; Thermo Fisher Scientific, Waltham, MA) and separated on an EASY-Spray PepMap RSLC C18 column (3 μm, 100 Å, 75 μm ID x 25 cm; Thermo Fisher Scientific) with mobile phase A (0.1% aqueous formic acid) and mobile phase B (0.1% aqueous formic acid /acetonitrile (20:80, v/v)) at a flow rate of 300 µl/min. MS spectra were recorded in data-dependent mode with a resolution of 120,000 and a cycle time of 3 s between master scans over an m/z range of 375–1,500 Da. Fragmentation was induced by higher energy collisional dissociation at a collision energy of 35 eV.

#### Data processing and analysis

Data processing and analysis were performed as previously described [37, 41]. Briefly, MS data (raw. files) were converted to centroid data (mzML format) using the MSconvert tool of the ProteoWizard program (version 3.0.1957; Palo Alto, CA). The mzML files were then analyzed using OpenMS in combination with the open-source software platform KNIME® [42]. Peptides were identified using combined search engines [41]. The OpenMS PeptideIndexer node was used to index peptide sequences with a defined leucine/isoleucine equivalence. The Protein Inference Analysis (PIA) technique was then used to predict proteins using the default parameters provided by the developers [43]. Protein abundances were determined using the FeatureFinderMultiplex node to generate spectral features, PIA-assisted FDR multiple score estimation and filtering (combined FDR score 0.01), ID mapping and combination with peptide IDs, and subsequent alignment, grouping, and normalization (i.e., MapAlignerIdentification, FeatureUnlabeledQT, and ConsensusmapNormalizer nodes) [44]. The OpenMS ProteinQuantifier node was then used to calculate label-free quantification (LFQ) of proteins and peptides based on the intensities of the three most abundantly detected peptides. The corresponding LFQ output files were read as CSV output tables and exported to Microsoft Office Excel. Downstream analyses were performed with proteins with no absent values in all samples.

Volcano plots were generated using VolcaNoseR (https://huygens.science.uva.nl/VolcaNoseR2/) [45]. To label relevant proteins in the volcano plot, we comprehensively examined all significantly dysregulated proteins by using PubMed’s advanced search option for title/abstract and specific tags, depending on the analyzed tissues. For eWAT, “white adipose tissue” OR “inflammation” OR “insulin resistance” AND “protein symbol” were used. In this case of the aorta, the tags “aorta” OR “inflammation” OR “atherosclerosis” OR “vasculature” AND “protein symbol” were selected. Functional analyses including Gene Ontology (GO) and Ingenuity Pathway Analysis (IPA) (QIAGEN, Venlo, Netherlands) were performed based on the whole proteome or differentially expressed proteins (p < 0.05), respectively. GO was determined with the Database for Annotation, Visualization, and Integrated Discovery (DAVID; NIAID, Bethesda, MD) platform. Network analysis was performed with Cytoscape software (Version 3.9.1, Seattle, WA).

### Quantification of plasma MCP-1

Plasma MCP-1 was analyzed using a commercially available ELISA kit following the manufacturer’s instructions (#EA-2408-SO, BioCat, Heidelberg, Baden-Wurttemberg, Germany). Samples were diluted 1:4.

### Immunophenotyping of peripheral blood

Peripheral blood was collected by facial vein puncture into EDTA-coated collection tubes. Samples were analyzed using a fully automated hematology analyzer (V-Sight; Menarini Diagnostics, Florence, Italy). For flow cytometry, 50 µl of blood were treated with ACK buffer to lyse red blood cells, and Fc receptors were blocked with anti-mouse CD16/CD32 (BD Biosciences, Franklin Lakes, NY). Subsequently, samples were stained with CD45-FITC, CD11b-AlexaFluor647, and Ly6G-APC-Cy7 antibodies (BD Biosciences) and analyzed using a Cytoflex LX (Beckman Coulter Life Sciences, Brea, CA). Data were acquired using CytExpert software (Beckman Coulter Life Sciences) and the analysis was performed using FlowJo (Treestar Inc., San Carlos, CA).

### Measurements of aortic reactivity

Relaxation to cumulatively increasing concentrations of acetylcholine (Ach) and the nitric oxide (NO) donor sodium nitroprusside (SNP) was recorded in vessels preconstricted to 80% of the maximal KCl (60 mmol/l)-induced contraction using U-46,619 (#538,944; Merck, Darmstadt, Germany) as a stable analog of thromboxane A2 as described [46, 47]. Relaxation values were expressed as a percentage of the initial U-46,619-induced contraction.

### Histological analyses of aortas and aortic valve sections

Mice were anesthetized by i.v. injection of Na-pentobarbital (200 mg/kg body weight), perfused with PBS/EDTA for 15 min, and then with 10% neutral buffered formalin for 15 min. The thoracic part of the aorta was excised and stored in formalin until oil red O (ORO) staining was performed to quantify the atherosclerotic lesions by *en face* analysis. The upper two-thirds of the heart were fixed in formalin for 24 h and stored in 30% sucrose until aortic valves sectioning. Staining, imagining, and quantification of the aortas and aortic valve sections were performed as previously described [48].

### Electron microscopy

Aortic tissue was fixed in 2.5% (wt/vol) glutaraldehyde and 2% (wt/vol) paraformaldehyde in 0.1 M cacodylate buffer (pH 7.4) for 2 h and post-fixed in 2% (wt/vol) OsO_4_ for 2 h at RT. After dehydration in graded series of ethanol, tissues were infiltrated (ethanol and TAAB epoxy resin, pure TAAB epoxy resin) and placed in TAAB epoxy resin (8 h), transferred into embedding moulds, and polymerized (48 h, 60 °C). Ultrathin Sect. (70 nm) were cut with a UC 7 Ultramicrotome (Leica Microsystems, Vienna, Austria) and stained with lead citrate for 5 min and platin blue for 15 min. Electron micrographs were taken at 120 kV using a Tecnai G2 transmission electron microscope (FEI, Eindhoven, Netherlands) with a Gatan ultrascan 1000 CCD camera (− 20 °C; Digital Micrograph acquisition software; Gatan, Munich, Germany, and Serial EM).

### Biomechanics of the aorta

Immediately after isolation, aortic specimens were frozen in Ringer’s solution containing 10% DMSO. For the extension-inflation test, the samples were slowly thawed overnight and a segment with a length of 5 mm that was straight as possible. Two disposable blunt cannulas (Sterican 0.40 × 25 mm) were then inserted into both ends of the aortic sample with tweezers and fixed by knotting with surgical sutures. Cyanoacrylate adhesive glue (Loctite Super Kleber, Power Flex) was used to achieve a stronger fixation between the samples and the cannulas. Two black dots, each one-third the length of the sample from the cannulas, served as markers for the length measurements with the video extensometer (VE). The specimen (with the cannulas) was then inserted load-free into the extension-inflation test setup as previously described [49].

#### Preconditioning

The preconditioning ensured a reproducible behavior of the aorta under cyclic loading. In this procedure, the aortas were first stretched axially to 1.3 and then subjected to a pressure of 100 mmHg for 10 min. The aortas were then subjected to three pressure cycles from 0 to 100 mmHg. After preconditioning, the unloaded length L and the unloaded outer diameter D of the aorta were measured with the VE.

#### Extension-inflation test

The aortas were axially stretched in increments of 0.1 from 1.2 to 1.7, while the aorta was pressurized five times at 70, 90, and 110 mmHg at each axial stretch step. Extension-inflation tests were performed with continuous recording of axial force, transmural pressures, outer diameter, and axial gauge length. The axial inversion stretch (IVS), where axial force is independent of inflation pressure, is assumed to be consistent with the in vivo axial stretch of the aorta and was therefore determined for each sample. To determine the IVS, we used the relationships between axial force and length as well as between pressure and axial stretch, similar to methods described by others [49–51]. For comparative purposes, circumferential and axial stresses in the aortic wall were estimated assuming that the tubular samples were circular cylindrical, thin-walled, homogeneous, and incompressible [49]. The unloaded vessel thickness required to calculate the stresses was determined after testing in the gage region using the VE [50]. The axial and circumferential stresses were plotted against the corresponding stretches. Axial and circumferential stretches were calculated as l/L and d/D, respectively, where l is the loaded axial length and d as the loaded outer diameter, resulting in dimensionless values of the stretches.

### Association studies in humans

Serum samples were obtained from a cross-sectional study involving a total of 128 individuals (65 healthy volunteers and 63 patients with metabolic syndrome) aged 45 to 65 years. These samples and all corresponding biochemical parameters of the patients were collected as part of a previous study [52]. Serum MMP12 concentrations were measured using a quantitative sandwich enzyme immunoassay (#EK0950; Boster Bio, Pleasanton, CA) according to the manufacturer’s instruction and negative values were set to zero.

### Statistical analyses

Statistical analyses were performed using GraphPad Prism 9.3.1 software. Data are presented as mean ± SD or SEM (for myography) and differences between two groups were assessed using the unpaired t test. For the human samples, Mann-Whitney-U test was used instead because of skewed distributions. Correlations were calculated using Spearman’s method. Receiver operating characteristic (ROC) curve analysis was performed to evaluate the diagnostic ability of MMP12. The following levels of statistical significance were used: *p < 0.05, **p ≤ 0.01, ***p ≤ 0.001.

## Results

### MMP12 deficiency reduces circulating lipid concentrations and improves glucose tolerance and insulin sensitivity in LdlrKO mice

**Fig. 1:**
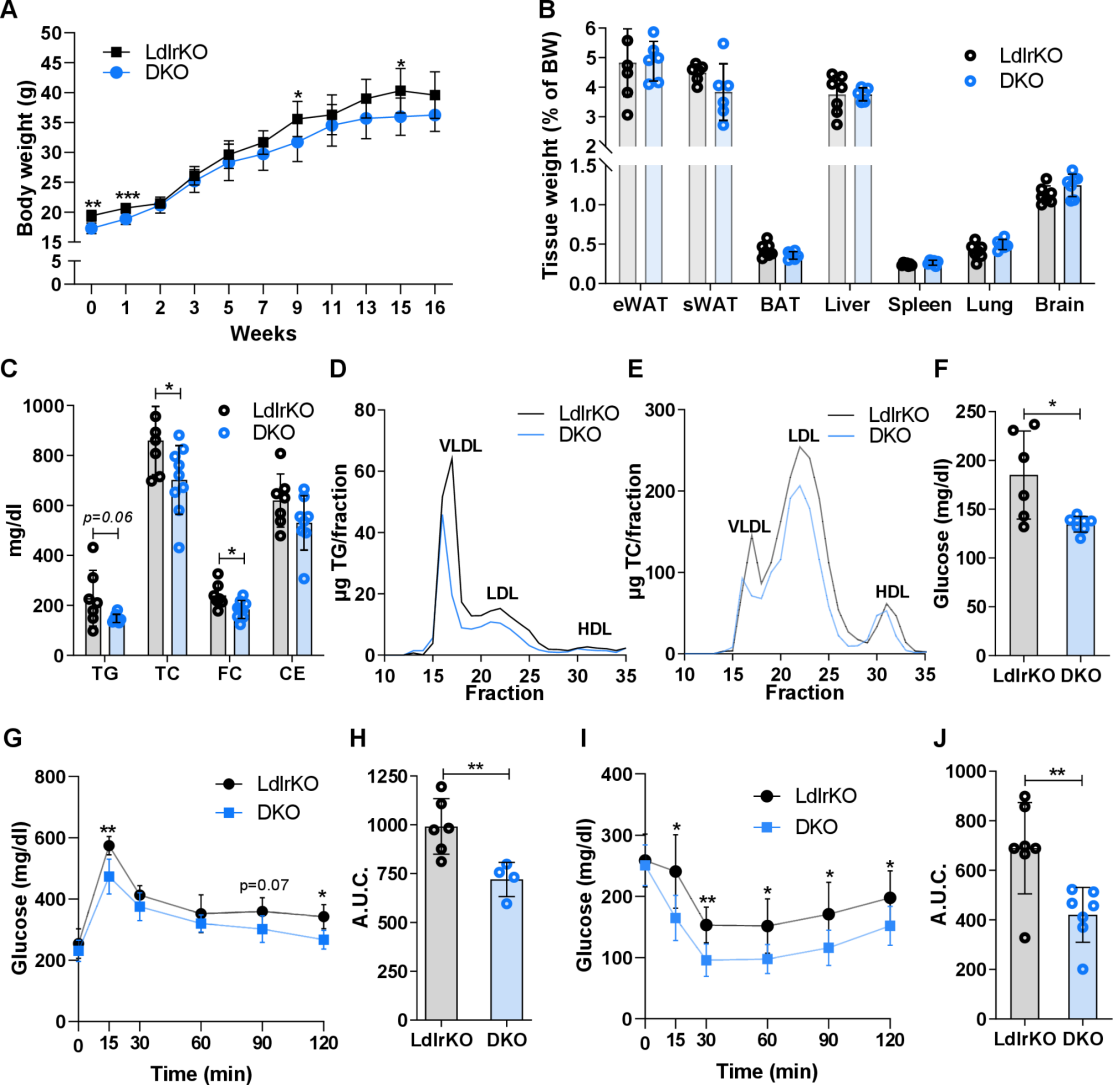
Reduced circulating lipid concentrations and improved glucose tolerance and insulin sensitivity in DKO mice. Male LdlrKO and DKO mice were fed HFSC for 16 weeks. **(A)** Body weight curves (n = 6–7) and **(B)** tissue weights relative to total body weight (n = 7). **(C)** Plasma triglyceride (TG), total cholesterol (TC), free cholesterol (FC), and cholesteryl ester (CE) concentrations in 12-h fasted mice (n = 7–9). Lipoprotein profiles of **(D)** TG and **(E)** TC in pooled plasma samples (n = 7–9) after fast protein liquid chromatography separation. **(F)** Plasma glucose concentrations in 12-h fasted mice (n = 6–7) and during **(G)** oral glucose tolerance (n = 4–6) and **(I)** insulin tolerance tests (n = 7). Area under the curve (A.U.C) calculated from **(H)** oral glucose tolerance and **(J)** insulin tolerance tests. All data represent mean values ± SD. *p < 0.05, **p ≤ 0.01

We first investigated whether constitutive deletion of MMP12 affected body or tissue weights upon a HFSC diet feeding for 16 weeks. DKO mice had slightly reduced body weight particularly before the dietary treatment, while tissue weights remained unchanged (Fig. 1A and B). At the end of the dietary treatment, we observed significant reductions in plasma TC and FC and a trend in TG levels (Fig. 1C) of 12 h-fasted DKO mice, with decreased TG (Fig. 1D) and TC (Fig. 1E) content in apolipoprotein B-containing lipoproteins as determined by fast protein liquid chromatography. Moreover, DKO mice displayed lower fasting plasma glucose concentrations (Fig. 1F) as well as improved glucose tolerance and insulin sensitivity (Fig. 1G-J). These data indicate that the genetic knockout of MMP12 leads to improvements in systemic metabolism.

### Reduced inflammation and formation of crown-like structures in eWAT of DKO mice

**Fig. 2:**
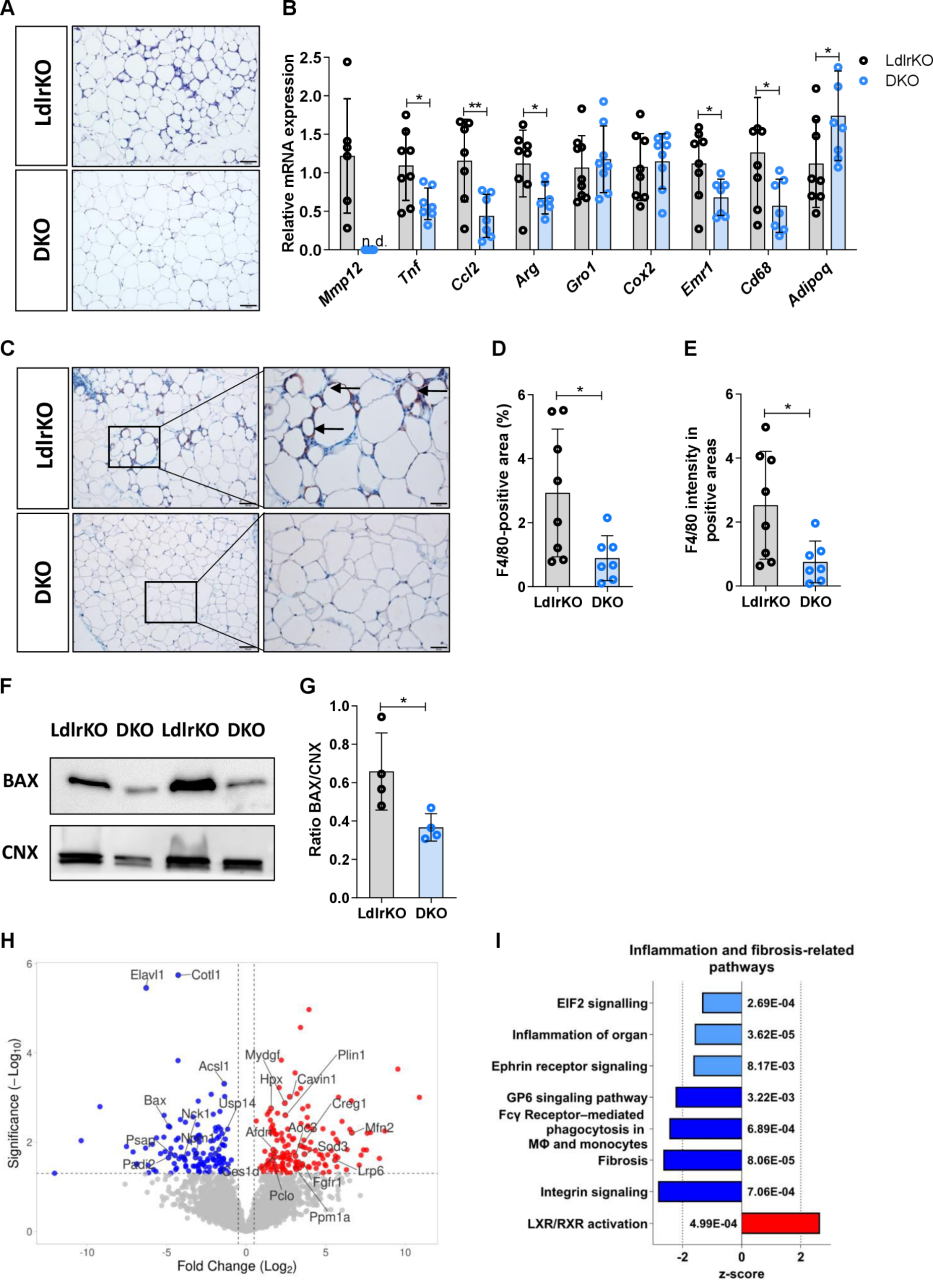
Reduced crown-like structure formation in DKO eWAT and downregulation of inflammatory and fibrosis-related pathways. **(A)** H&E staining of eWAT sections (scale bar, 100 μm). **(B)** mRNA expression of proinflammation genes and anti-inflammatory *Adipoq* in eWAT relative to *Rplp0* expression (n = 6–9). **(C)** F4/80 immunohistochemistry in eWAT sections (scale bar, 100 μm; insets: scale bar, 50 μm). Quantification of F4/80 immunohistochemistry in eWAT sections from LdlrKO and DKO mice (n = 8 − 7) with **(D)** percentage of positively stained areas and **(E)** mean intensity in the positive areas. **(F)** Protein expression of BAX in eWAT. Calnexin (CNX) was used as a loading control. **(G)** Densitometric quantification of BAX/CNX (n = 4). **(H)** Volcano plot showing selected upregulated (red) and downregulated proteins (blue) reported to play a role in either inflammation and/or fibrosis in eWAT. **(I)** Ingenuity Pathway Analysis (IPA) from untargeted proteomics data of eWAT showing downregulated gene sets (z-score < 2 in light blue; z-score > 2 in dark blue) and upregulated gene sets (red) in DKO mice. Figures represent data from 6 LdlrKO and 6 DKO mice. Data represent mean values ± SD. *p < 0.05, **p ≤ 0.01. n.d., not detected

Since WAT dysfunction plays a crucial role in the development of systemic insulin resistance and the onset of CMDs, we further investigated whether the genetic deficiency of MMP12 leads to morphological and/or functional changes in this tissue. Histologically, H&E staining revealed a substantial number of crown-like structures (CLS) in eWAT from LdlrKO but not from DKO mice (Fig. 2A) despite comparable adipocyte size (Additional file 2, Fig. S1A-B). In line, mRNA expression levels of the pro-inflammatory genes *Tnf*, *Ccl2*, *Arg1*, *Emr1*, and *Cd68* were reduced, whereas the expression of the anti-inflammatory and insulin-sensitizing marker *Adipoq* was increased (Fig. 2B). Reduced F4/80 staining for macrophages in eWAT sections from DKO mice corroborated the lower abundance of CLS, a histological hallmark of WAT inflammation (Fig. 2C-E), indicating that macrophage infiltration is impaired upon the genetic deletion of MMP12. Because CLS formation is directly linked to adipocyte death, we also investigated whether genetic knockout of MMP12 affects apoptosis in these cells. Accordingly, we observed reduced protein expression of the pro-apoptotic regulator BAX in eWAT from DKO mice (Fig. 2F and G). To confirm our data on a large scale, we performed shotgun proteomics on eWAT from LdlrKO and DKO mice. Of the 2756 proteins quantified (Additional file 3), 274 were differentially expressed in eWAT from DKO mice (p < 0.05). Among the 129 downregulated proteins (Log_2_FC < -0.5), ELAVL1, PADI1, BAX, PSAP, COTL1, NCK1, NPM1, USP14, and ACSL1 (Fig. 2H and Additional file 1, Table S2) as well as among the 145 upregulated proteins (Log_2_FC > 0.5), HPX, PCLO, AFDN, CES1D, MYDGF, PLIN1, CAVIN1, AOC3, PPM1A, CREG1, FGFR1, SOD3, LRP6, and MFN2 (Fig. 2H and Additional file 1, Table S3) were associated with WAT inflammation and/or insulin resistance, as identified with a Pubmed search. The up- and downregulated proteins were then classified into molecular functions, biological processes, and cellular components using GO analysis (Additional file 2, Fig. S2A-C and Additional file 3). In addition, network analyses revealed that proteins related to inflammation, fatty acid β-oxidation, PPAR lipids, lipid binding, mitochondrion, thermogenesis, and ECM were significantly dysregulated in DKO eWAT (Additional file 2, Fig. S2D). The canonical pathways in IPA showed downregulation of fibrosis-related and inflammatory pathways as well as the activation of the LXR/RXR pathway in eWAT from DKO mice, suggesting an anti-inflammatory and pro-resolving phenotype (Fig. 2I and Additional file 3). Our results suggest that the genetic knockout of MMP12 improves WAT function in CMDs by changing its proteome signature toward a healthier phenotype, thereby ameliorating local inflammation, apoptosis, and CLS formation.

### Reduced low-grade systemic inflammation and altered immune cell composition in DKO mice

**Fig. 3:**
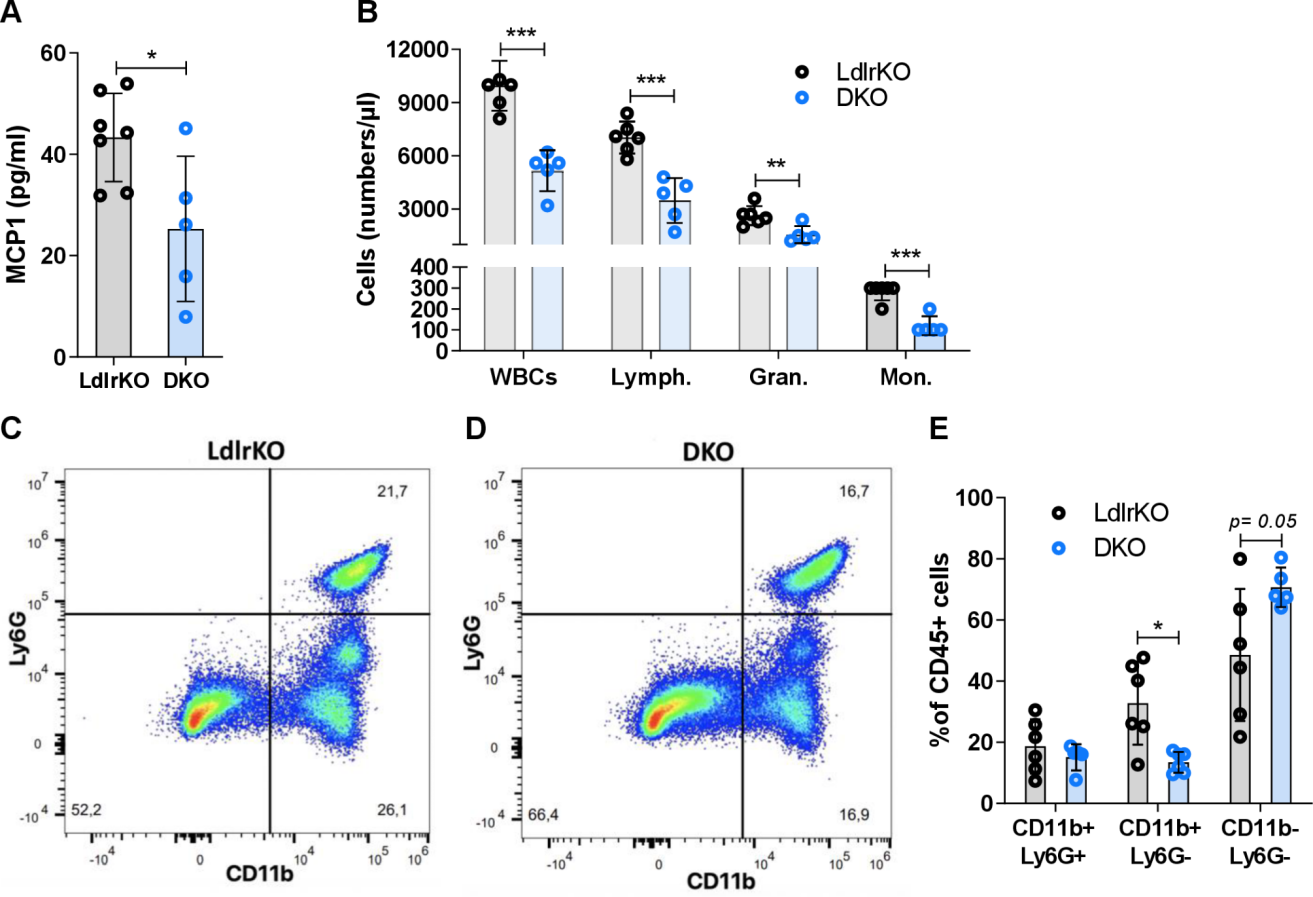
Reduced systemic inflammation and altered immune cell composition in blood of DKO mice. **(A)** Circulating MCP-1 concentrations in LdlrKO and DKO mice measured by ELISA (n = 5–7). **(B)** Absolute white blood cell count analyzed by a fully automated hematology analyzer (n = 5–6). **(C, D)** Representative flow cytometric plots of Ly6G vs. CD11b staining of CD45 + cells. **(E)** Quantification of Ly6G vs. CD11b staining of immune cells in peripheral blood (n = 5–6). Data represent mean values ± SD. *p < 0.05, **p ≤ 0.01, ***p ≤ 0.001

Considering that CMDs impair immune function, we examined whether MMP12 deficiency also affects systemic inflammation and the composition of the white blood cell (WBC) population. Concentrations of monocyte chemoattractant protein 1 (MCP-1) were reduced in the plasma of DKO mice (Fig. 3A). In line, total WBC counts were decreased in DKO mice due to a reduction in all major cell populations (lymphocytes, granulocytes, and monocytes) (Fig. 3B). To complement the blood cell count measurements, we analyzed the composition of circulating immune cells via flow cytometry. After separation of all CD45^+^ cells to exclude non-hematopoietic cells, we separated CD45^+^ cells based on their CD11b and Ly6G expression. We detected a lower number of CD11b^+^Ly6G^−^ cells in the peripheral blood of DKO mice (Fig. 3C-E). This finding suggests that the genetic deletion of MMP12 alleviates systemic inflammation and alters immune cell composition.

### Improved vasodilatory capacity and biomechanical properties in aortas from DKO mice

**Fig. 4:**
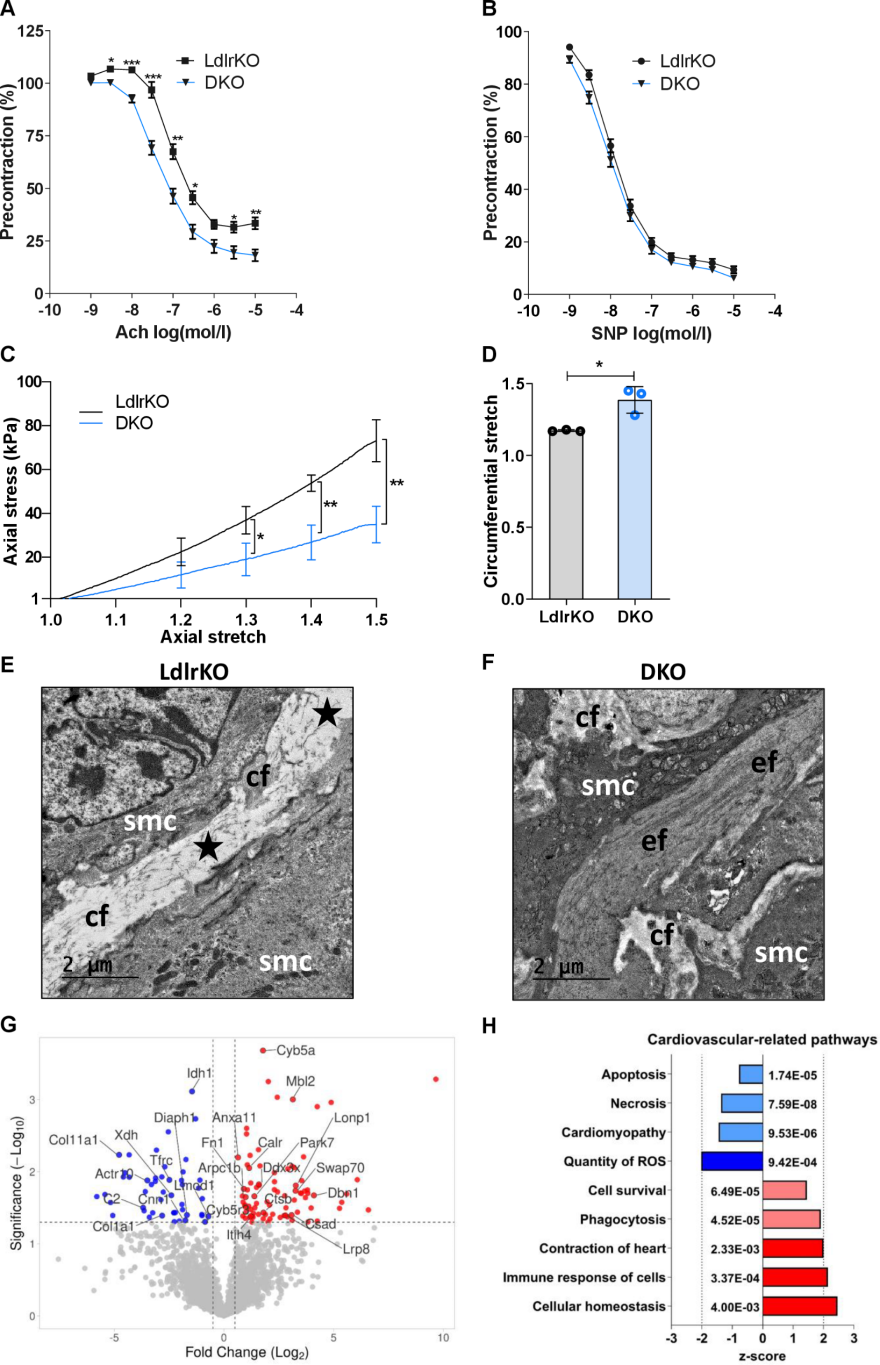
Improved cardiometabolic features in DKO mice. Mouse aortic rings preconstricted with U-46,619 were treated with cumulative addition of **(A)** acetylcholine (Ach) or **(B)** sodium nitroprusside (SNP) to induce relaxation. Relaxation values are expressed as percentage of the initial U-46,619-induced contraction. Data represent mean values ± SEM of 19–20 aortic rings per genotype isolated from 5 LdlrKO and 5 DKO mice; *p < 0.05, **p ≤ 0.01, ***p ≤ 0.001. **(C)** Axial stress versus axial stretch behavior (n = 3) and **(D)** circumferential stretch at 100 mmHg intramural pressure (n = 3). Data represent mean values ± SD. *p < 0.05; ** p ≤ 0.01. Representative transmission electron micrographs from the aortic arch of **(E)** LdlrKO and **(F)** DKO mice. **(E)** Residuals of elastic fibers (asterisk), smooth muscle cells (smc), and collagen fibers (cf.) in LdlrKO mice. **(F)** Normal elastic fibers (ef), smooth muscle cells, and collagen fibers in close proximity to elastic fibers in DKO mice. Scale bars, 2 μm. **(G)** Volcano plot showing selected upregulated (red) and downregulated proteins (blue) in DKO aortas reported to play a role in either inflammatory processes, atherogenesis, and/or vascular dysfunction in aortas. **(H)** Ingenuity Pathway Analysis (IPA) from nontargeted proteomics data of aortas showing downregulated protein sets (z-score < 2 in light blue; z-score > 2 in dark blue) and upregulated gene sets (red) in DKO mice. Figures represent data from 6 LdlrKO and 6 DKO mice

Systemic inflammation and immune cell dysregulation also play a central role in the development of CVD, including atherosclerosis. Since endothelial dysfunction represents the first step in atherogenesis, we investigated whether genetic loss of MMP12 impacts vasorelaxation by myography of U-46,619 preconstricted aortic rings in response to cumulatively increasing concentrations of Ach and SNP, which mediate endothelium and direct smooth muscle cell relaxation, respectively. We observed an improved relaxation pattern in aortic rings from DKO mice after exposure to Ach (Fig. 4A) but not to SNP (Fig. 4B). These results indicate that the genetic knockout of MMP12 improves the vasodilatory capacity of the vascular wall by ameliorating endothelial cell function without affecting smooth muscle cell responsiveness.

Since MMP12 is an ECM protein that primarily degrades elastin, we hypothesized that MMP12 deficiency might also lead to improved elasticity and biomechanical properties of the atherosclerotic arterial wall. We assessed the axial and circumferential stretch of thoracic aortas, which revealed a marked improvement of aortic stiffness in DKO mice (Fig. 4C), with a significantly softer axial mechanical behavior at axial stretches > 1.2. Given that the axial inversion stretches of the mouse aortas studied ranged from 1.3 to 1.75, differences in axial mechanical properties between DKO and LdlrKO mice at axial prestretches in vivo are to be expected. The circumferential stretch of the LdlrKO aortas (1.17 ± 0.01) was significantly lower than the corresponding stretch of the DKO mice (1.39 ± 0.09) (Fig. 4D), indicating a softer mechanical behavior of DKO aortas also in the circumferential direction. In line, electron microscopy revealed only remnants of elastic fibers that appeared to be replaced by collagen in aortas from LdlrKO mice (Fig. 4E), whereas the elastic fibers remained intact in the aortas from DKO animals (Fig. 4F). Overall, the mechanical behavior in the axial and circumferential directions and the electron micrographs suggest that genetic deletion of MMP12 results in better preservation of elastic fibers, which translates into diminished arterial stiffness of the atherosclerotic vessel.

### Altered protein expression and regulation of cardiovascular-related pathways in aortas from DKO mice

To identify proteins and pathways dysregulated upon genetic deletion of MMP12, we performed untargeted proteomics on aortas from LdlrKO and DKO mice. A total of 2231 proteins were quantified (Additional file 4), of which 132 were significantly dysregulated in aortas from DKO mice (p < 0.05). Among the 49 downregulated proteins (Log_2_FC < -0.5), COL11A1, C2, ACTR10, COL1A1, TFRC, XDH, CNN1, DIAPH1, IDH1, CYB5R3, and LMOD1 (Fig. 4G and Additional file 1, Table S4) were associated with vascular inflammation and/or atherosclerosis, as identified by a Pubmed search. The 83 upregulated proteins (Log_2_FC > 0.5) included DBN1, LONP1, SWAP70, MBL2, CSAD, LRP8, CTSB, CYB5A, DDX3X, PARK7, ITIH4, CALR, ARPC1B, FN1, and ANXA11 associated with the selected tags (Fig. 4G and Additional file 1, Table S5). The up- and downregulated proteins were then assigned into molecular functions, biological processes, and cellular components using GO analysis (Additional file 2, Fig. S3A-C). Network analyses showed that proteins associated to ECM, endocytosis, cytoskeleton, and positive regulation of the immune system were the most altered pathways in DKO aortas (FDR < 0.05, Additional file 2, Fig. S3D). Additionally, functional analyses utilizing the IPA software revealed several cardiovascular-related pathways to be dysregulated in DKO aortas, such as “Quantity of ROS” as the most inhibited and “Cellular homeostasis” as the most activated pathway (Fig. 4H). Thus, our data indicate that genetic deletion of MMP12 leads to multiple changes in the vascular wall, which may protect the mice from cardiovascular disease, including atherosclerosis.

### Reduced atherosclerotic plaque size and collagen deposition in aortic valve sections of DKO mice

**Fig. 5:**
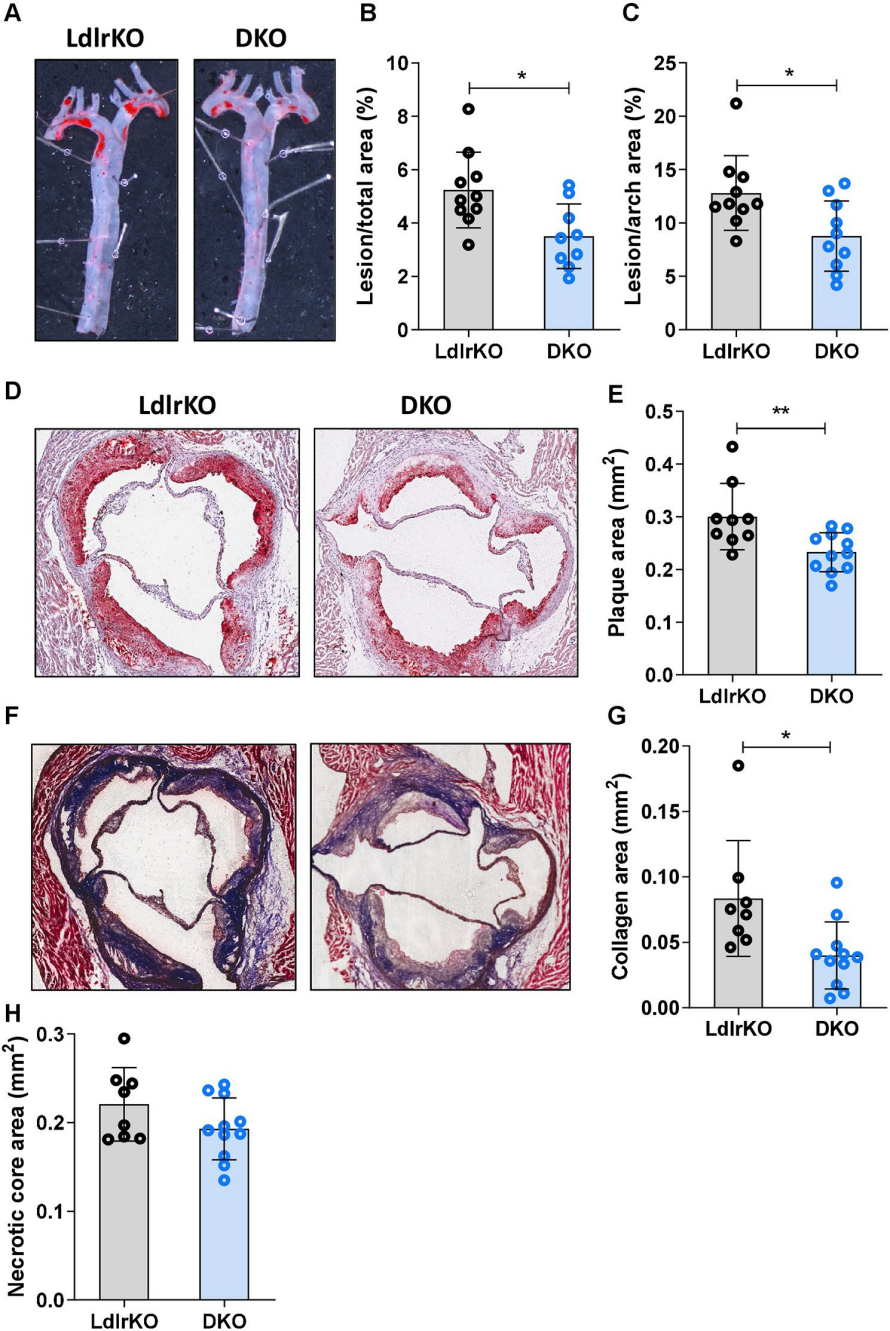
Reduced atherosclerotic plaque size and collagen content in aortas of DKO mice. Female LdlrKO and DKO mice were fed a HFSC diet for 20 weeks. **(A)** Oil red O staining of *en face* aortas and quantification of atherosclerotic lesions in the **(B)** total aorta and **(C)** aortic arch. **(D)** Oil red O staining of aortic valve sections (magnification, 5X) and **(E)** plaque area quantification. **(F)** Masson´s Trichrome staining of aortic valve sections (magnification, 5X) and quantification of **(G)** collagen-positive area and **(H)** necrotic core. Data represent mean values (n = 8–11) ± SD. *p < 0.05; ** p ≤ 0.01

We next determined whether genetic deletion of MMP12 affects atherogenesis. After 20 weeks of feeding with HFSC diet, DKO mice had reduced plaque size in the thoracic aorta (LdlrKO: 5.24 ± 1.42%; DKO: 3.50 ± 1.21%; p = 0.016) (Fig. 5A and B) and aortic arch area (LdlrKO: 12.81 ± 3.49%; DKO: 8.77 ± 3.29%; p = 0.01) (Fig. 5A and C). This was consistent with the decreased lesion size in aortic valve sections of DKO mice, as evidenced by reduced ORO staining (LdlrKO: 0.30 ± 0.06 mm^2^; DKO: 0.23 ± 0.04 mm^2^; p = 0.007) (Fig. 5D and E). Due to the prolonged dietary treatment and in agreement with a more advanced plaque phenotype [53], we failed to visualize macrophages by MoMa-2 staining (Additional file 2, Fig. S4). Accordingly, qPCR of proinflammatory markers showed high Ct values in both genotypes (Additional file 1, Table S6). Since fibrous cap formation is a defining feature of advanced atherosclerotic lesions, we performed Masson’s Trichrome staining to detect collagen content and necrotic core as the acellular compartment within the lesion. While collagen content was significantly reduced in aortic valve sections from DKO mice (LdlrKO: 0.08 ± 0.04 mm^2^; DKO: 0.04 ± 0.02 mm^2^; p = 0.015, Fig. 5F and G), the necrotic core size was comparable between the genotypes (LdlrKO: 0.22 ± 0.04 mm^2^; DKO: 0.19 ± 0.03 mm^2^; p = 0.13) (Fig. 5F and H). These data indicate that genetic knockout of MMP12 attenuates atherosclerosis by reducing plaque size and altering plaque composition.

### Circulating MMP12 is weakly associated with metabolic syndrome and clinical and laboratory indicators for CMDs in humans

**Fig. 6:**
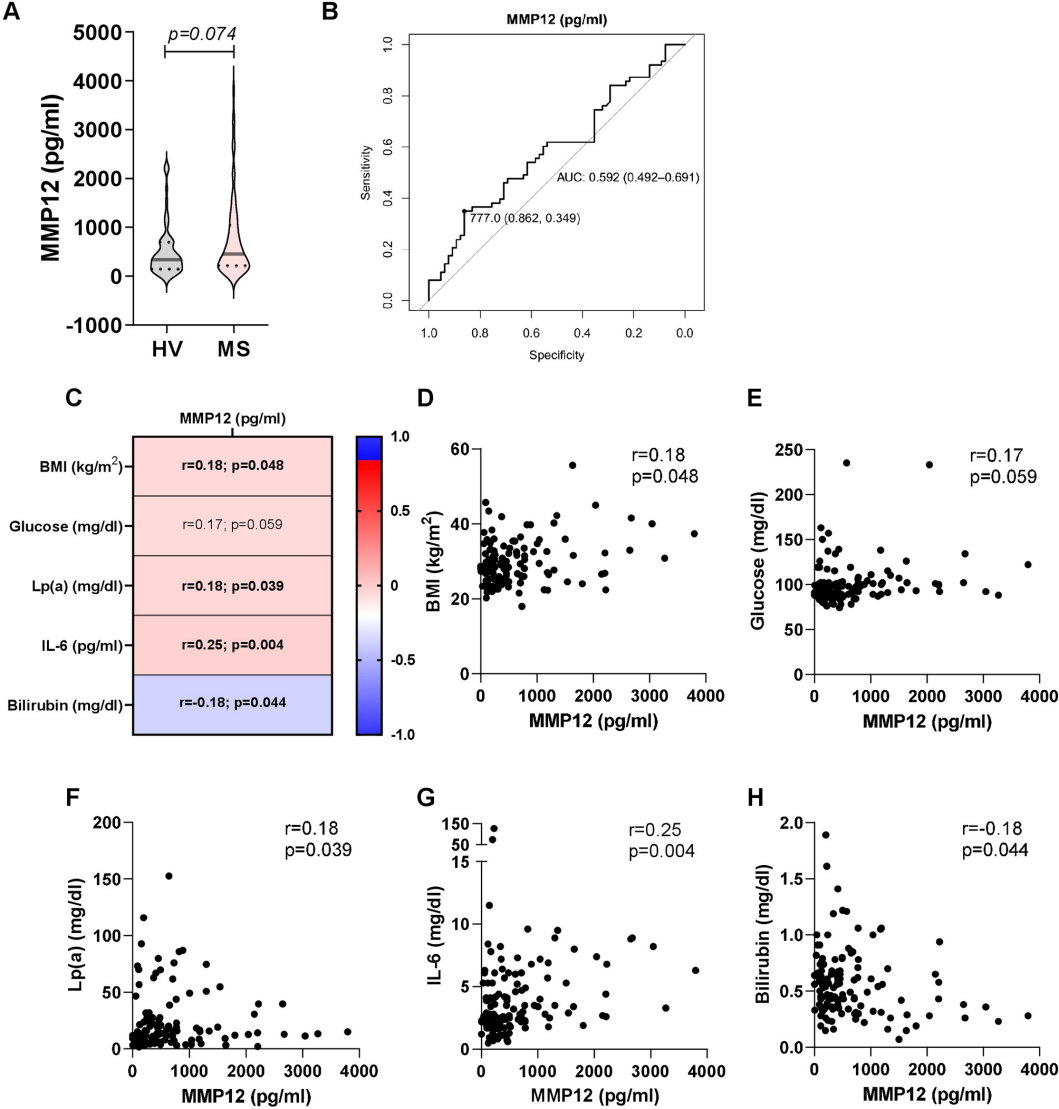
Correlations of circulating MMP12 with clinical and laboratory indicators for cardiometabolic diseases **(A)** Violin plots of MMP12 concentrations in serum from healthy volunteers (HV) and from patients with metabolic syndrome (MS). Groups were compared using the Mann-Whitney U test (n = 65 − 63). **(B)** Receiver operating characteristic (ROC) curve for the prediction of MS using MMP12. The graph shows the ROC curve, area under the curve (AUC), 95% confidence interval, and the best cut-off value according to Youden index along with specificity and sensitivity. **(C)** Heat map for correlation analyses of serum MMP12 concentrations with body mass index (BMI), as well as serum levels of glucose, lipoprotein (a) (Lp(a)), interleukin 6 (IL-6), and bilirubin levels. Correlations were quantified using Spearman’s correlation coefficient (r). Correlation plots of circulating MMP12 concentrations (pg/ml) with **(D)** BMI (kg/m^2^), **(E)** glucose (mg/dl), **(F)** Lp(a) (mg/dl), **(G)** IL-6 (mg/dl), and **(H)** bilirubin (mg/dl) (n = 128)

To determine the relevance of MMP12 in human disease, we measured its levels in serum from patients with metabolic syndrome (MS), who had 41% higher concentrations of circulating MMP12 compared to healthy volunteers (HV) (Fig. 6A). ROC curve analysis to evaluate the accuracy of MMP12 in predicting MS revealed an area under the curve of 0.592 with a 95% confidence interval of 0.492–0.691 (Fig. 6B). Based on the cut-off value (777 pg/ml) according to the Youden index, the specificity and sensitivity were 86.2% and 34.9%, respectively, and the negative and positive predictive values were 57.7% and 71.0%, respectively. We further assessed possible associations between MMP12 and clinical and laboratory indicators of CMD in humans. Spearman correlations revealed that serum MMP12 concentrations were only weakly positively associated with body mass index (BMI), as well as serum levels of glucose, lipoprotein (a) (Lp(a)), and interleukin-6 (IL-6) and a minor negative association was observed between MMP12 and bilirubin (Fig. 6C-H). The observed poor discriminatory capacity of circulating MMP12 for MS and the weak association with indicators for CMDs do not support the usefulness of circulating MMP12 as a marker of CMD in humans.

## Discussion

MMP12 is a macrophage-secreted protein that has been previously associated with pro-inflammatory functions in a variety of pathological conditions in humans and in animal models [16, 17, 21, 23, 54]. Several studies have also addressed its role in individual pathologies encompassed by CMDs such as insulin resistance or CVDs [27, 30–32]. However, many important aspects of its specific contribution to the pathogenesis of CMDs remain either inconsistent or elusive. Therefore, we investigated the effects of genetic MMP12 deficiency in a cardiometabolic mouse model that mimics the human CMD pathology by simultaneously developing corresponding comorbidities. Here, we demonstrated that genetic deletion of MMP12 improved the cardiometabolic phenotype, altered immune cell composition, ameliorated low-grade systemic inflammation and WAT function, improved insulin resistance, and reduced atherosclerosis development.

DKO mice displayed improved glucose tolerance and insulin sensitivity despite comparable body and tissue weights to control animals. This observation is consistent with a previous study showing that Mmp12KO mice are more insulin sensitive than wild type mice after 10 weeks of high-fat diet feeding [31]. However, it contradicts a report in which high-fat diet-fed Mmp12KO mice had comparable glucose tolerance or insulin sensitivity to control animals [32]. However, the control animals used in the latter study did not develop persistent obesity and glucose intolerance. Our findings show that MMP12 deficiency on the LdlrKO background protects from the development of systemic metabolic disturbances.

Proteomic profiling of eWAT and aortas as key tissues involved in the onset of CMDs helped to identify proteins and signaling pathways affected under genetic Mmp12-deficient conditions. In eWAT from DKO mice, we detected protein upregulation of a plethora of local protective factors (such as CES1D, MYDGF, PLIN1, CAVIN, PPM1A, CREG1, FGFR1, SOD3, and MFN2) against metabolic dysregulation, inflammation, reprogramming of the diabetic gene signature in WAT and other tissues including non-alcoholic fatty liver disease, impairment of brown adipose tissue thermogenesis, and in atherosclerosis [55–66]. Several proteins downregulated in eWAT, such as PADI2, PSAP, NCK1, and NPM1, with proven pro-inflammatory properties [67–70] that are normally secreted into the extracellular space, have been linked to the progression of atherosclerosis [68, 71–73]. It is therefore conceivable that their downregulation might lead to several beneficial effects in the vascular wall of DKO mice. The pro-apoptotic regulator BAX was among the most downregulated proteins, supporting our hypothesis that the genetic knockout of MMP12 improves WAT function by alleviating local inflammation and CLS formation. In agreement, DKO mice had decreased concentrations of circulating MCP-1, which mediates monocyte recruitment to WAT and contributes to macrophage infiltration, inflammation, and ultimately insulin resistance [74].

Several studies have shown that obesity and prolonged high-fat diet feeding enhance systemic inflammation, hematopoiesis, and lymphopoiesis [75–77]. The mechanism by which the genetic deletion of MMP12 reduced WBC counts and altered immune cell number and composition remains elusive. We speculate that MMP12 affects immune cell survival by acting as a transcription factor [78], modifying the immune response via enzymatic processing of chemokines [79] or generating chemotactic elastin-derived peptides that are liberated due to its elastolytic capabilities [80, 81]. These bioactive molecules with signaling capabilities counteract elastin degradation. Thus, elastin-derived peptide formation might prevent and/or delay atherosclerosis and T2D [82]. In line, proteomic analyses of aortas from DKO mice revealed upregulation of anti-inflammatory and atheroprotective markers and vasorelaxation mediators (ITIH4, LRP8/APOER2, MBL2, DBN1, PARK7/DJ-1) [83–87], whereas proteins related to the progression of atherosclerosis (DIAPH1) [88], foam cell formation and macrophage ferroptosis (IDH1) [89], and endothelial dysfunction and reactive oxygen species (ROS) production (XDH) [90] were downregulated. Thus, differential abundance of proteins associated with vasorelaxation and ROS production, downregulation of the ROS pathway, and myography data indicate that MMP12 deficiency improves endothelial dysfunction, which is considered an early stage in the development of atherosclerosis. Genetic knockout of MMP12 attenuated axial stiffening of carotid arteries during aging [91], consistent with the improved mechanical properties of atherosclerotic aortas in extension-inflation tests, intact elastin fibers, and a reduced abundance of COL11A1 and COL1A1 in DKO mice. In contrast, LdlrKO mice had only remnants of elastic fibers that might have been replaced by collagen fibers, and increased elastin degradation and collagen deposition may contribute to arterial stiffness and vascular calcification [92].

Published data from humans, rabbits, or other mouse models have already described detrimental effects of MMP12 on atherosclerosis development [27, 28, 30, 93]. Consistently, our data in DKO mice corroborate atheroprotective effects of MMP12 loss by reduced atherosclerotic plaque and lesion size in aortas and aortic valve sections, respectively. These changes might be explained by the decrease in circulating lipid levels, particularly the reduction of the pro-atherogenic apolipoprotein B-containing lipoproteins and the number and composition of WBCs. However, no effects on plasma lipid parameters or WBC counts or composition, which play a pivotal role in atherogenesis, were described in a transgenic MMP12 rabbit model or in MMP12 inhibitor studies [27, 30]. Given the decreased number of WBCs in the circulation, one might expect attenuated monocyte infiltration into the arterial wall and thus lower macrophage content in DKO mice. However, due to the prolonged dietary treatment and consistent with a more advanced plaque phenotype [53], we failed to detect macrophages in atherosclerotic valve sections from either group of mice by MoMa-2 staining and aortic gene expression analysis.

Taken together, our data in mice suggest that MMP12 is an attractive target for controlling and even ameliorating CMDs. MMP12-selective inhibitors have already been successfully tested in mouse models of allergic asthma, progression of osteoarthritis, and atherosclerosis [27, 94, 95]. In addition, researchers have recently described a novel anti-MMP12 approach based on a pro-drug that is selectively cleaved by its own target (i.e., MMP12) to release its own inhibitor [96]. Blocking MMPs to treat various diseases, however, should be considered with caution: (i) MMPs are very complex molecules with both beneficial and detrimental functions under physiological or pathological conditions [97]. (ii) Due to the high sequence identity of MMPs, the development of specific inhibitors is challenging, but detailed prediction of specific MMP12 inhibitors using recent machine learning approaches may help with this issue [98].

In the present study, circulating MMP12 was only weakly associated with indicators of CMDs in humans. Of note, serum MMP12 levels partially reflect MMP12 bioavailability in tissues, where MMP12 exerts its biological functions. Despite the poor capacity of circulating MMP12 concentrations for serving as a marker for CMDs, it does not exclude its (patho)physiological role in humans.

## Conclusion

We conclude that the genetic deletion of MMP12 in a mouse model mimicking CMDs in humans leads to altered immune cell composition and improvements in systemic inflammation, insulin sensitivity, and atherosclerotic development, supported by beneficial changes in the proteome signature of WAT and aortas. In humans, circulating MMP12 levels are weakly linked to MS and indicators of CMDs. Further studies are needed to establish the association between tissue MMP12 bioavailability and CMDs.

### Electronic supplementary material

Below is the link to the electronic supplementary material.

Supplementary Material 1

Supplementary Material 2

Supplementary Material 3

Supplementary Material 4

## Data Availability

The mass spectrometry proteomics dataset(s) supporting the conclusions of this article have been deposited to the ProteomeXchange Consortium via the PRIDE repository [99], with the dataset identifier PXD046532 in http://www.ebi.ac.uk/pride/archive/projects/PXD046532.

## Abbreviations

Ach: acetylcholine
BMI: body mass index
CE: cholesteryl esters
CLS: crown-like structure
CMD: cardiometabolic disease
CVD: cardiovascular disease
DKO: Mmp12-deficient mice on the Ldlr-deficient background
ECM: extracellular matrix
eWAT: epididymal white adipose tissue
FC: free cholesterol
GTT: glucose tolerance test
GO: gene ontology
HFSC: high-fat sucrose- and cholesterol-enriched
IL-6: interleukin-6
IPA: ingenuity pathway analysis
ITT: insulin tolerance test
IVS: inversion stretch
Lp(a): lipoprotein a
MCP-1: monocyte chemoattractant protein 1
NO: nitric oxide
ORO: Oil red O
ROC: receiver operating characteristic
ROS: reactive oxygen species
SNP: sodium nitroprusside
TG: triglyceride
TC: total cholesterol
T2D: type 2 diabetes
VE: video extensometer
WBCs: white blood cells
