# Supplementary material for: Genetic deletion of MMP12 ameliorates cardiometabolic disease by improving insulin sensitivity, systemic inflammation, and atherosclerotic features in mice
Source: Cardiovasc Diabetol. 2023 Nov 28;22:327. doi: 10.1186/s12933-023-02064-3 (PMC10685620; doi:10.1186/s12933-023-02064-3)
Supplement: Supplementary file 1 — Supplementary Material 1 [file 12933_2023_2064_MOESM1_ESM.pdf]

Table S1: Primers used for quantitative real-time PCR

Gene	Forward primer	Reverse primer
Mmp12	CTGCTCCCATGAATGACAGTG	AGTTGCTTCTAGCCCAAAGAAC
Rplp0	AGATTCGGGATATGCTGTTGGC	TCGGGTCCTAGACCAGTGTTC
Tnfa	CCACCACGCTCTTCTGTCTAC	AGGGTCTGGGCCATAGAACT
Ccl2	TTAAAAACCTGGATCGGAACCAA	GCATTAGCTTCAGATTTACGGGT
Arg	TGGCTTGCGAGACGTAGAC	GCTCAGGTGAATCGGCCTTTT
Gro1	CTGGGATTCACCTCAAGAACATC	CAGGGTCAAGGCAAGCCTC
Cox2	TGAGCAACTATTCCAAACCAGC	GCACGTAGTCTTCGATCACTATC
Emr1	CTTTGGCTATGGGCTTCCAGTC	GCAAGGAGGACAGAGTTTATCGTG
Cd68	AACAGGACCTACATCAGAGC	TCAAGGTGAACAGCTGGAGA
Adipoq	GGAGATGCAGGTCTTCTTGG	CGAATGGGTACATTGGGAAC
Mcp5	ATTTCCACACTTCTATGCCTCCT	ATCCAGTATGGTCCTGAAGATCA
Il6	CTGCAAGAGACTTCCATCCAG	AGTGGTATAGACAGGTCTGTTGG
Il1b	GAAATGCCACCTTTTGACAGTG	TGGATGCTCTCATCAGGACAG

Table S2: Fold change in Log2 and p-value of selected downregulated proteins in eWAT of DKO mice

Protein symbol	Protein name	Log_2FC	p-value
ELAVL1	ELAV like RNA binding protein 1	-6.29	3.496E-06
PADI2	Peptidyl arginine deiminase, type II	-4.89	2.223E-02
BAX	BCL2-associated X protein	-4.88	4.363E-03
PSAP	Prosaposin	-4.48	1.972E-02
COTL1	Coactosin like F-actin binding protein 1	-4.27	1.805E-06
NCK1	NCK adaptor protein 1	-3.93	2.176E-02
NPM1	Nucleophosmin 1	-3.18	2.204E-02
USP14	Ubiquitin specific peptidase 14	-1.92	1.788E-02
ACSL1	Acyl-CoA synthetase long-chain family member 1	-1.38	4.900E-04

Table S3: Fold change in Log2 and p-value of selected upregulated proteins in eWAT of DKO mice

Protein symbol	Protein name	Log2FC	p-value
MFN2	Mitofusin 2	6.64	6.145E-03
LRP6	Low density lipoprotein receptor-related protein 6	5.58	2.522E-02
SOD3	Superoxide dismutase 3, extracellular	3.66	1.858E-02
FGFR1	Fibroblast growth factor receptor 1	3.53	3.089E-02
CREG1	Cellular repressor of E1A-stimulated genes 1	3.41	1.195E-02
PPM1A	Protein phosphatase 1A, magnesium dependent, alpha isoform	3.15	4.063E-02
AOC3	Amine oxidase, copper containing 3	3.10	2.046E-02
CAVIN1	Caveolae associated 1	2.74	9.590E-04
PLIN1	Perilipin 1	2.46	2.484E-03
MYDGF	Myeloid derived growth factor	2.43	1.335E-03
CES1D	Carboxylesterase 1D	1.79	2.145E-02
AFDN	Afadin, adherens junction formation factor	1.70	1.475E-02
PCLO	Piccolo (presynaptic cytomatrix protein)	1.66	2.535E-02
HPX	Hemopexin	1.56	1.478E-03

Table S4: Fold change in Log2 and p-value of selected downregulated proteins in aortas of DKO mice

Protein symbol	Protein name	Log_2FC	p-value
COL11A1	Collagen, type XI, alpha 1	-4.78	5.833E-03
C2	Complement C2	-3.68	3.152E-02
ACTR10	ARP10 actin-related protein 10	-3.49	1.320E-02
COL1A1	Collagen, type I, alpha 1	-2.82	4.062E-02
TFRC	Transferrin receptor	-2.49	1.300E-02
XDH	Xanthine dehydrogenase	-2.40	2.121E-02
CNN1	Calponin 1	-1.76	2.121E-02
DIAPH1	Diaphanous related formin 1	-1.69	4.725E-02
IDH1	Isocitrate dehydrogenase 1 (NADP+), soluble	-1.45	7.696E-04
CYB5R3	Cytochrome b5 reductase 3	-0.86	4.954E-02
LMOD1	Leiomodin 1 (smooth muscle)	-0.71	4.171E-02

Table S5: Fold change in Log2 and p-value of selected upregulated proteins in aortas of DKO mice

Protein symbol	Protein name	Log_2FC	p-value
DBN1	Drebrin 1	4.10	2.126E-02
LONP1	lon peptidase 1, mitochondrial	3.27	1.555E-02
SWAP70	SWA-70 protein	3.26	1.838E-02
MBL2	Mannose-binding lectin (protein C) 2	3.14	9.937E-04
CSAD	Cysteine sulfinic acid decarboxylase	3.08	3.989E-02
LRP8	LDR receptor-related protein 8, apolipoprotein e receptor	2.80	4.032E-02
CTSB	Cathepsin B	1.80	3.688E-02
CYB5A	Cytochrome b5 type A (microsomal)	1.78	2.085E-04
DDX3X	DEAD box helicase 3, X-linked	1.59	1.500E-02
PARK7	Parkinson disease (autosomal recessive, early onset) 7	1.39	2.189E-02
ITIH4	Inter alpha-trypsin inhibitor, heavy chain 4	1.22	4.129E-02
CALR	Calreticulin	1.15	8.883E-03
ARPC1B	Actin related protein 2/3 complex, subunit 1B	0.99	2.245E-02
FN1	Fibronectin 1	0.90	1.728E-02
ANXA11	Annexin A11	0.64	6.333E-03

Table S6: Average Ct values of pro-inflammatory markers in aortas after 20-week feeding of HFSC diet

Gene	LdlrKO	DKO
Ccl2	31.87	31.96
Il1b	31.12	30.50
Cox2	28.85	28.98
Mrc5	31.55	32.31
Tnf	>40	>40
Il6	>40	>40
