# Supplementary material for: Genetic deletion of MMP12 ameliorates cardiometabolic disease by improving insulin sensitivity, systemic inflammation, and atherosclerotic features in mice
Source: Cardiovasc Diabetol. 2023 Nov 28;22:327. doi: 10.1186/s12933-023-02064-3 (PMC10685620; doi:10.1186/s12933-023-02064-3)
Supplement: Supplementary file 2 — Supplementary Material 2 [file 12933_2023_2064_MOESM2_ESM.pdf]

Supplementary Figure 1

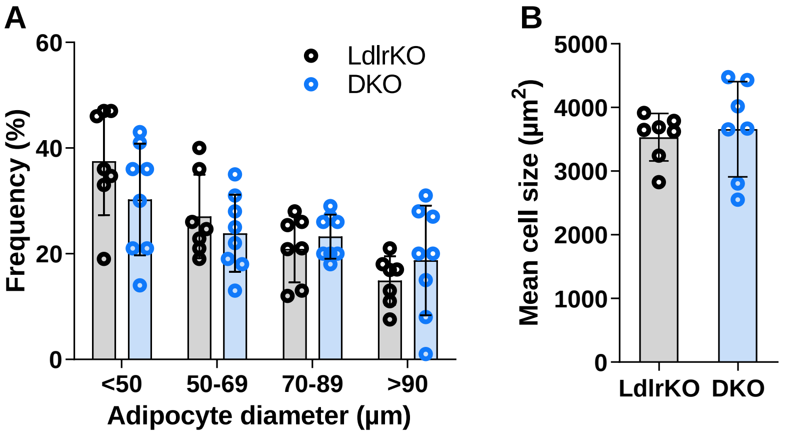

Figure S1. Unchanged adipocyte size in eWAT of DKO mice after 16 weeks of HFSC feeding. (A) Distribution of adipocyte diameters and **(B)** mean adipocyte size from eWAT quantified from H&E stainings (n = 7).

Supplementary Figure 2

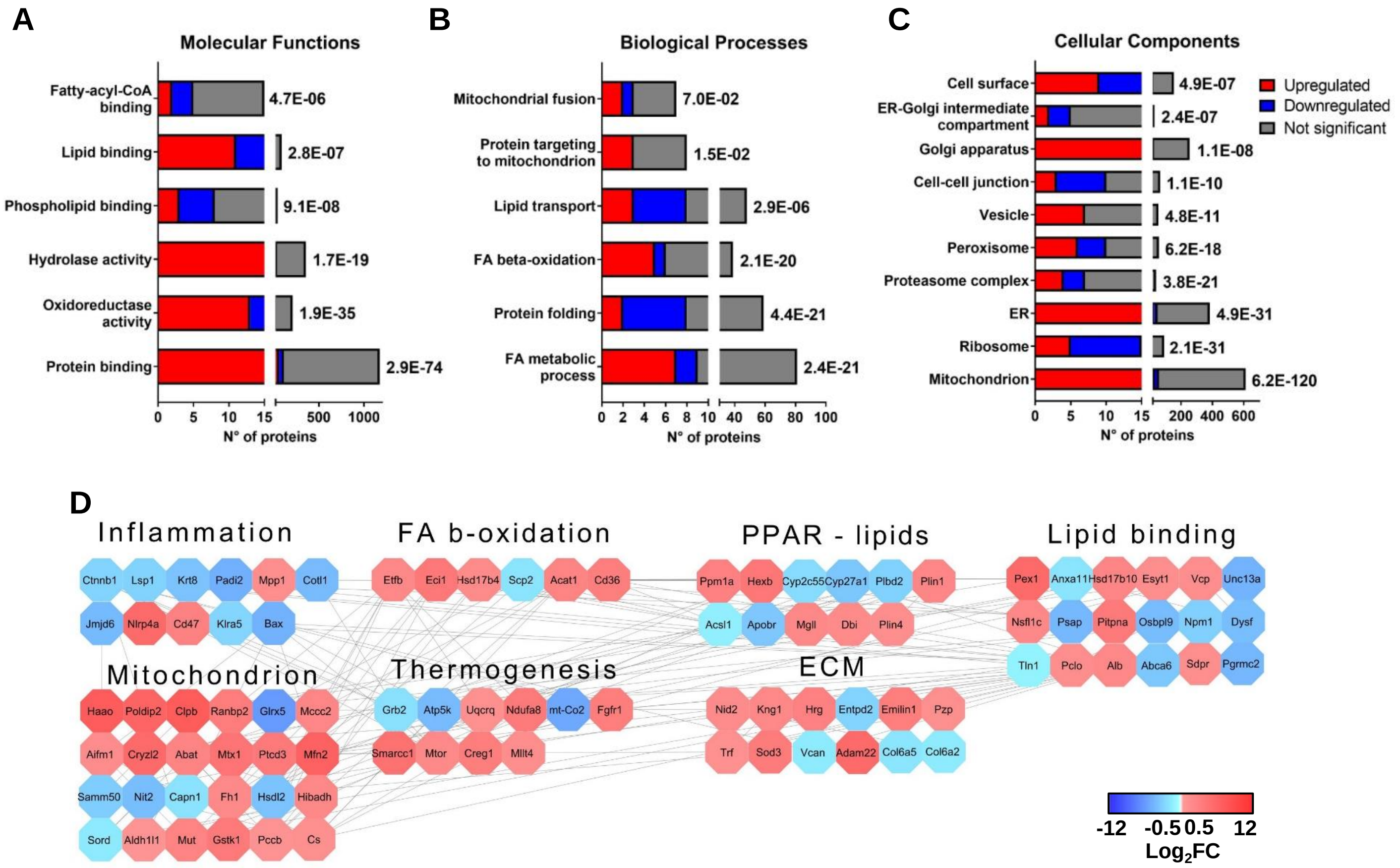

Figure S2. MMP12 deletion is associated with changes in protein expression in eWAT of DKO mice. Male LdlrKO and DKO mice were fed HFSC for 16 weeks. Significantly dysregulated proteins were classified into **(A)** Molecular Functions, **(B)** Biological Processes, and **(C)** Cellular Components by Gene Ontology (GO) analysis. p-values are indicated to the right of the bars. Up- and downregulated proteins in eWAT of DKO mice are shown in red and blue, respectively. **(D)** Network analysis and pathway enrichment for significant proteins ($p < 0.05$) created with Cytoscape software. Figures represent data from 6 LdlrKO and 6 DKO mice.

Supplementary Figure 3

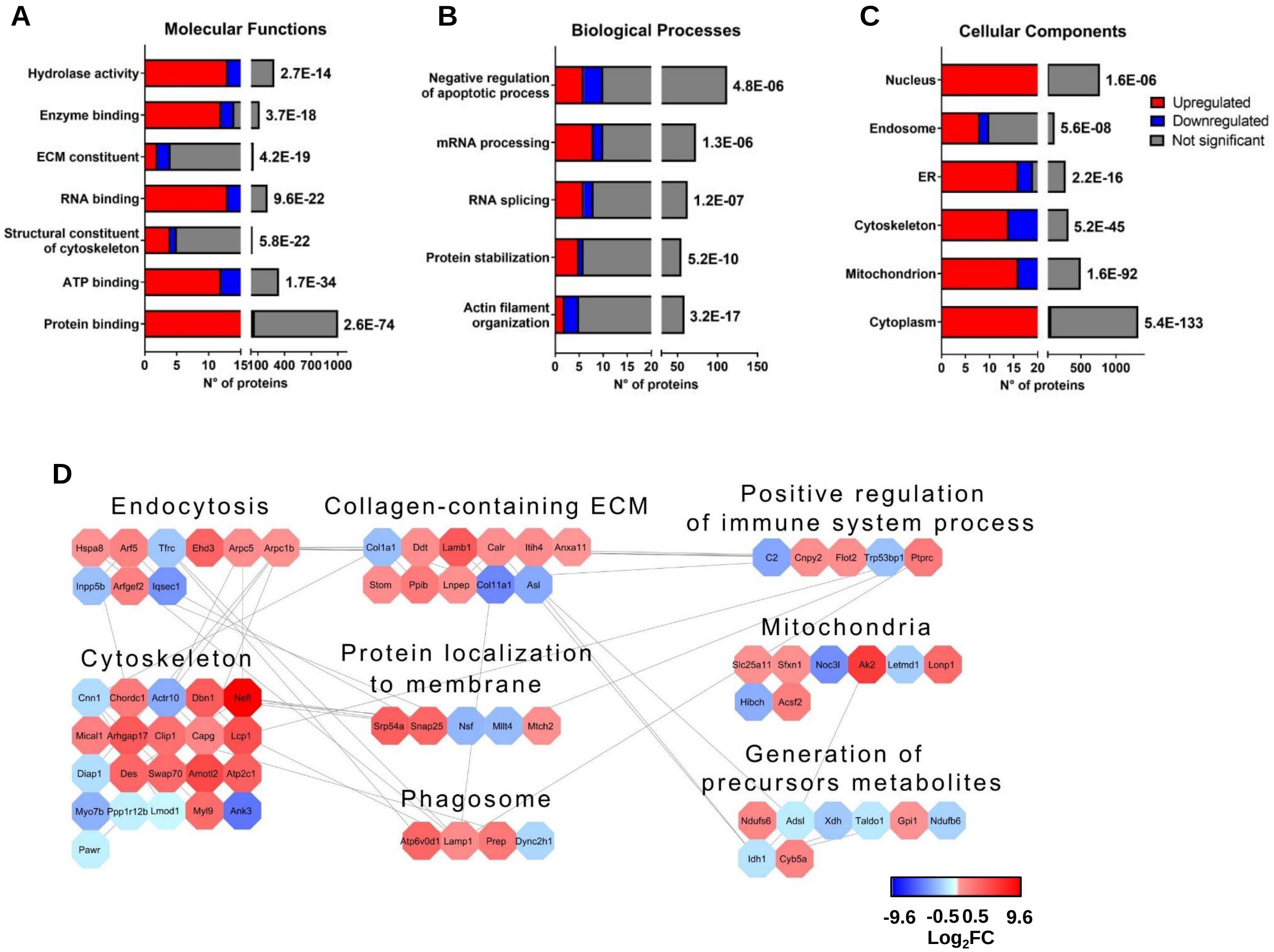

Figure S3. MMP12 deletion is associated with differential protein expression in aortas of DKO mice. Male LdlrKO and DKO mice were fed HFSC for 16 weeks. Significantly dysregulated proteins were classified into **(A)** Molecular Functions, **(B)** Biological Processes, and **(C)** Cellular Components by gene ontology (GO) analysis. p-values are indicated to the right of the bars. Up- and downregulated proteins in aortas of DKO mice are shown in red and blue, respectively. **(D)** Network analysis and pathway enrichment for significant proteins ($p < 0.05$) created with Cytoscape software. Figures represent data from 6 LdlrKO and 6 DKO mice.

Supplementary Figure 4

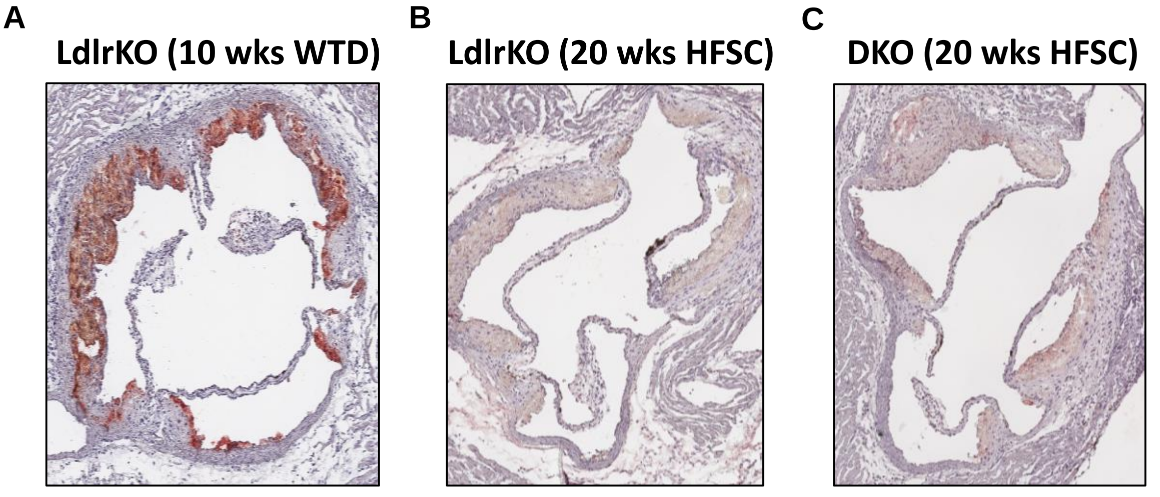

Figure S4. Macrophage staining (MoMa-2) in aortic valve sections of LdlrKO and DKO mice. Representative images of MoMa-2 immunohistochemical staining of aortic valve sections from **(A)** LdlrKO mice after 10-week feeding with Western type diet (WTD) (positive control), **(B)** LdlrKO and **(C)** DKO mice after 20-week feeding with HFSC diet (magnification, 5X).
